# Oxidative Stress and Intestinal Transcriptome Changes in *Clostridium perfringens* Type A-Caused Enteritis in Deer

**DOI:** 10.3390/genes16080949

**Published:** 2025-08-11

**Authors:** Meihui Wang, Qingyun Guo, Zhenyu Zhong, Qingxun Zhang, Yunfang Shan, Zhibin Cheng, Xiao Wang, Yuping Meng, Yulan Dong, Jiade Bai

**Affiliations:** 1 Laboratory of Veterinary Anatomy, College of Veterinary Medicine, China Agricultural University, Beijing 100193, China; wangmeihui718@163.com; 2 Beijing Milu Ecological Research Center, Beijing 100076, China; guoqingyun1987@126.com (Q.G.); miluhome@126.com (Z.Z.); zhangqingxun1990@126.com (Q.Z.); shanyunfang@yeah.net (Y.S.); czb@milupark.org.cn (Z.C.); 3 National Conservation and Research Center for Milu, Beijing 100076, China; 4 Institute of Zoology, Chinese Academy of Sciences, Beijing 100101, China; theowx@outlook.com; 5 Beijing Acadamy of Science and Technology, Beijing 100089, China; mengyuping@bjast.ac.cn

**Keywords:** *Clostridium perfringens* type A, enteritis, oxidative stress, immunity, transcriptome

## Abstract

Background: *Clostridium perfringens* (*C. perfringens*) type A is a major cause of enteritis in farmed and wild deer populations, leading to significant economic losses in the deer industry. This bacterium produces toxins that damage the intestine. Methods: In this study, we performed transcriptome analysis by establishing an intestinal circulation model of the intestines of fallow deer (*Dama Dama*) inoculated with *C. perfringens* type A versus those not inoculated with *C. perfringens* type A. In a further step, we determined the protein content of immunoinflammation-related molecules by ELISA and the antioxidant capacity of the intestine to investigate the molecular mechanisms of *C. perfringens* type A-induced enteritis. Results: Transcriptome analysis revealed significant enrichment of pathways related to the haematopoietic system, oxidative stress, the immune system and intestinal tight junctions. Additionally, *C. perfringens* α-toxin enters the intestine and may be recognized by TLR6, activating the immune system, increasing the secretion of various cytokines and inflammasome components, inducing oxidative stress and damaging the intestine. Conclusions: This study provides a comprehensive transcriptomic basis for understanding the selective differential expression of genes in deer enteritis induced by *C. perfringens* type A and provides a broader guide for finding therapeutic approaches to deer enteritis.

## 1. Introduction

In deer breeding production, *Clostridium perfringens* (*C. perfringens*) enteritis is of concern due to its rapid onset, high mortality rate and economic losses. Infections are more frequent in the intestines of fit young and strong deer, and the incidence is higher in males than in females [1]. Clinical studies revealed that *C. perfringens* infections are very common in deer, and the isolates are mainly type A, C, D and E [2,3,4]. Deer haemorrhagic enteritis, also known as deer enteritis, is caused by the proliferation of *C. perfringens* in the intestinal tract. *C. perfringens* spreads through the intestinal tract of the deer, leading to acute toxaemia, with clinical signs of diarrhoea, convulsions, paralysis and sudden death [5,6,7]. As ruminant livestock, deer are an important part of the agricultural sector in terms of food, hunting and other products [8]. Therefore, deer health, with a specific focus on enteritis caused by *C. perfringens*, requires additional research attention.

*C. perfringens* is a gram-positive, anaerobic bacterium that is widely distributed nature, especially in the gastrointestinal tracts of humans and animals [9]. It is an important pathogen that causes different forms of tissue damage through its ability to secrete a wide variety of toxins and enzymes. It is characterized by low incidence (2–8%) but high case fatality rates (up to 100%) [10]. As a result, it can cause a broad array of diseases in various vertebrates. Based on the type of toxin production and pathogenicity, *C. perfringens* can be classified into seven types from A to G [11]. In general, α-toxin is present in all bacteria, and some bacteria produce enterotoxins that can cause food poisoning. Type B is defined by the production of β-toxin and ε-toxin. In addition, type C produce β-toxin, type D produces ε-toxin, type E produces ι-toxin, type F produces *C. perfringens* enterotoxin and type G produces necrotic enteritis B-like toxin [9]. These bacteria are capable of causing intestinal diseases in humans and animals, including food poisoning, necrotizing enterocolitis and enteritis [12]. Even though *C. perfringens* is generally present in the intestine as part of the gut microbiome, in pathological situations, this bacterium can proliferate abnormally, releasing toxins that either act locally or are absorbed into the bloodstream with severe effects on the host [13,14].

*C. perfringens* acts through the production of toxins, and the study of the effect of *C. perfringens* toxins is of particular importance to clarify the enteritis caused by *C. perfringens* in deer. *C. perfringens* α-toxin (CPA) is a major virulence factor during *C. perfringens* infection [15] and induces erythrocyte haemolysis in various species by inhibiting erythrocyte differentiation and impairing erythropoiesis [16]. The CPA is a zinc metalloenzyme composed of 370 amino acids that can traverse the cell membrane into the host in the presence of calcium ions [17]. It first hydrolyses phosphatidylcholine (PC) and sphingomyelin (SM) in the plasma membrane and triggers different pathways depending on the cell type involved. For example, in equine erythrocytes, PC cleavage is mainly performed by the intrinsic CPA phospholipase activity [18], whereas sheep erythrocytes containing only trace amounts of PC mainly activate SM metabolism after being affected by CPA [19]. In neutrophils, CPA activates endogenous phospholipase C and phosphorylates PI3K, which in turn phosphorylates PKCθ, MEK1/2, ERK1/2 system and NF-κB, resulting in inflammation and oxidative stress [16]. In other words, the toxin cause further damage to the intestinal barrier and promotes acute exacerbation of inflammation, oxidative stress and apoptosis. This damage leads to a loss of normal intestinal function, resulting in fluid and electrolyte excretion. However, there are huge genetic variations behind this complex process that require additional analysis.

In this study, we aimed to construct an intestinal loop model using milu as a model to investigate the structural and functional changes caused by intestinal inflammation induced by *C*. *perfringens* type A infection in milu. Additionally, transcriptomic analysis was used to reveal the molecular mechanisms underlying *C*. *perfringens* type A-induced intestinal inflammation in deer, with a focus on its regulatory effects on intestinal barrier function, immune inflammatory pathways and the oxidative stress system, thereby identifying new targets for clinical intervention.

## 2. Materials and Methods

### 2.1. Bacterial Strain and Growth Media

*C. perfringens* type A strain, isolated from milu (*Elaphurus davidianus*), was obtained from the Beijing Milu Ecological Research Center, and PCR detection of the virulence factors they carried only allowed the CPA gene (Forward: GCTAATGTTACTGCCGTTGA; Reverse: CCTCTGATACATCGTGTAAG) to be detected. It was grown overnight in Fluid Thioglycollate (FTG) medium (Solarbio, Beijing, China) at 37 °C in an anaerobic cabinet (Boxun Industrial Co., Ltd., Shanghai, China). The *C. perfringens* culture was incubated anaerobically overnight at 37 °C in 10 mL of FTG; then, the bacterial pellets were resuspended in 1 mL of culture supernatant and set aside.

### 2.2. Animal and Preparation of Loops

The animal experiments were carried out with approval from the Ethics Review Committee of Experimental Animal Welfare and Animal Experimentation of China Agricultural University (approval no. CAU202208112). One 2-month-old male fallow deer was used for the trial. The deer had not been vaccinated against *C. perfringens* type A and no previous cases of enteritis had been diagnosed in the herd. To establish haemorrhagic enteritis caused by *C. perfringens* type A, the deer was transferred to a separate animal experimental unit on the day before the experiment and deprived of food and water.

**Figure 1:**
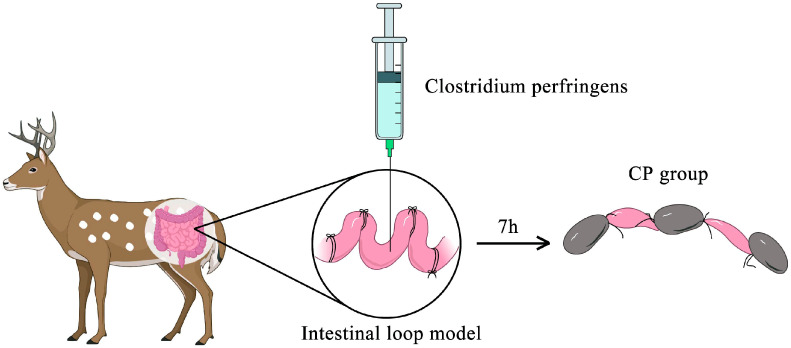
Process of *Clostridium perfringens* infection in an intestinal loop model. The jejunum of the deer is exposed by laparotomy and separated by ligation, leaving an empty segment of intestine between the loops. An equal amount of logarithmic growth phase *C. perfringens* culture was injected into each intestinal loop. At the end of the procedure, the intestinal loops were placed back into the abdominal cavity and the abdominal incision was closed with sutures to the muscle and skin, respectively. After 7 h, the intestine was removed again and the *C. perfringens*-injected intestinal segment appeared congested (brown intestinal loops in the figure) as CP group.

Anaesthesia was performed with xylazine hydrochloride injection (Huamu Animal Health Products Co., Ltd., Jilin, China), and subsequently, a dissection was performed via the linea mediana ventralis and the small intestine was exposed. The intestinal loops were about 3 cm in length and were securely ligated with medical sutures (Jinhuan, Shanghai, China). And a 5 cm gap was left between each of the two intestinal loops to avoid interfering with the blood supply. The intestine was rinsed with saline prior to injection, and 1 mL of FTG or *C. perfringens* type A (logarithmic growth period, approximately 1.5 × 10^8^ CFU/mL) culture was prepared as specified in Section 2.1 and injected in equal amounts into each intestinal loop (Figure 1). The three intestinal loops injected with FTG were named as controls. The three intestinal loops injected with *C. perfringens* type A were labelled as the CP group. When injecting the loops, care was taken to avoid over-distension of the loops. Then, the abdominal wall incisions were sutured to the muscle and skin separately to prevent the loss of body temperature, and the animal was deeply anaesthetized throughout the experiment. Seven hours after inoculation, anaesthesia was administered to the animals using xylazine hydrochloride injection, the abdominal cavity was reopened and the small intestine was removed in the order of inoculation. Finally, the deer was euthanized.

After gross analysis, the intestinal tissues were rapidly dissected and rinsed in PBS. Then, part of each loop of intestinal tissues was stored in a −80 °C refrigerator for transcriptional analysis, mRNA analysis and protein analysis. Another part of each loop was transferred to 10% formalin for histological examination.

### 2.3. Morphologic Analysis

Tissues were removed after 24 h of fixation in 10% formalin solution, rinsed and subjected to histopathological analysis. Intestinal tissue samples after dehydration with ethanol were embedded in paraffin and cut into 5 μm slices. The slices were then stained with a haematoxylin and eosin staining kit (Lab-Test Biotechnology, Beijing, China) and sealed with neutral gum. Finally, photomicrographs were taken using an Aperio CS2 scanner (Leica Biosystems, Wetzlar, Germany).

### 2.4. RNA Sequencing

Total RNA was isolated from the intestinal segments using Trizol. The intestinal segments were three duplicate samples from the control group and *C. perfringens* group. The samples of transcription analysis were sequenced by Biomarker Technologies, China (Beijing). After sequencing with Illumina NovaSeq 6000 (Illumina, San Diego, CA, USA), the Clean Data of each sample reached 5.78 GB, and the percentage of Q30 bases was 93.75% and above. Sequence assembly of the Clean Data was performed to obtain the unigene library of the species in Trinity software (version 2.15.0). Complete unigene splicing and functional annotation followed as well as unigene expression quantification, differential analysis and functional enrichment analysis in Diamond software (version 3.12). The relevant RNAseq data is accessed in NCBI and the sample was submitted under accession number PRJNA946184.

### 2.5. RNA-Seq Data Analysis

The criteria for screening the DEGs were FDR < 0.05 and Fold Change ≥ 2 in the biomarker cloud platform. Then, GO enrichment and KEGG pathway analyses were performed with the “clusterProfiler”, “cowplot” and “org. Hs.eg.db” packages (*p*-Value < 0.05 and q-Value < 0.05). Furthermore, heatmaps were generated using “pheatmap” package.

### 2.6. Protein–Protein Interaction (PPI) Network Construction

The PPI network of identified DEGs was constructed using the STRING online database (http://string-db.org, accessed on 8 August 2024). Functional modules in interaction networks were identified using the Markov clustering algorithm. The most stringent protein interaction screening criteria were applied (confidence > 0.9).

### 2.7. qPCR Validation

To quantify the expression of each mRNA in the intestine, real-time SYBR-Green PCR analysis was performed. Total RNA was isolated with TRIzon Reagent (Thermo Fisher Scientific, Waltham, MA, USA) to remove DNA contamination. The RNA concentration and purity were measured with a NanoDrop One (Thermo Fisher Scientific, Waltham, MA, USA). Afterwards, the total RNA was mixed with HiScript QRTsupermix for qPCR (+gDNA wiper) (Vazyme, Nanjing, China) to synthesize cDNA. The synthetic cDNA was diluted 10-fold and PCR was performed on an ABI SimpliAmp PCR instrument (Applied Biosystems, Foster City, CA, USA). Primers were synthesized by Sangon Biotech (Shanghai, China) Co., Ltd. qPCR was performed with SYBR green master mix (Vazyme, Nanjing, China) using the primers in Table 1. Changes in fluorescence were monitored on an ABI 7500 instrument (Applied Biosystems, Foster City, CA, USA).

### 2.8. ELISA

First of all, fresh lysis buffer (Cloud-Clone Crop., Wuhan, China) was used to measure the total protein extracted according to the kit instructions. Then the concentrations of IL-1β, IL-4, IL-6, IL-8, IL-22 and TNF-α (Cloud-Clone Crop., Wuhan, China) in the intestine were determined with the kit. The monitoring range of ELISA kit is 15.6~1000 pg/mL for IL-1β, 15.6~1000 pg/mL for IL-4, 7.8~500 pg/mL for IL-6, 15.6~1000 pg/mL for IL-8, 15.6~1000 pg/mL for IL-22 and 7.8~500 pg/mL for TNF-α.

### 2.9. Intestinal Antioxidant Capacity Test

CAT was detected in the intestine of the deer according to the kits provided by Nanjing Jiancheng Bioengineering Institute (Nanjing, China). GSH, T-AOC and MDA were detected in the intestine of the deer according to the kits provided by Beyotime Biotechnology Co. (Shanghai, China). According to the instructions of the kit, the intestinal proteins were extracted, and added to the assay working solution system, and then the absorbance of the samples was measured by an enzyme marker to calculate the CAT, GSH, T-AOC and MDA contents based on the corresponding equations.

### 2.10. Statistical Analyses

All experimental results were analyzed in SPSS 20.0 and presented in the form of mean ± standard error of mean (SEM). Figures were generated by GraphPad Prism 8.1 (San Diego, CA, USA) and R Studio (version 4.0.3). The differences between the experimental values of the two groups were assessed using Student’s t-test with the following statistical significance: * *p* < 0.05; ** *p* < 0.01; and *** *p* < 0.001.

## 3. Results

### 3.1. Histological Damage of C. perfringens Type A-Infected Intestine

**Figure 2:**
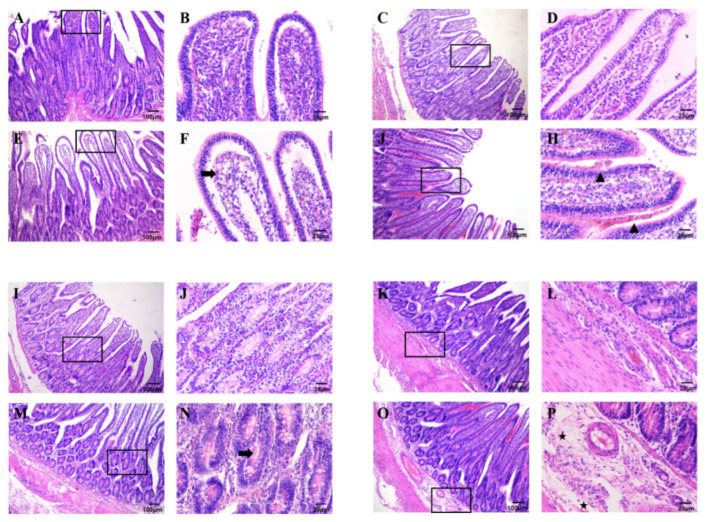
Histopathology of the intestinal tract after inoculation with *C. perfringens*. Haematoxylin–eosin staining showed intestinal structures. (**A**–**D**,**I**–**L**) Representative tissue sections of intestinal segments not inoculated with *C. perfringens*. The intestine was well organized and structurally hierarchical in the control group. (**E**–**H**) Representative histological sections of intestine injected with *C. perfringens* strain to the mucosal layer showing villi haemorrhage and necrosis. (**M**–**P**) Representative histological sections of the mucosal and submucosal layers of the intestine injected with *C. perfringens* strains, showing inflammatory cell infiltration in the intestine. The arrow represents necrosis, the triangle represents haemorrhage and the star represents inflammatory cell infiltration in figures. Size bars/magnifications: (**A**,**C**,**E**,**G**,**I**,**K**,**M**,**O**) = 100 μm/×100; (**B**,**D**,**F**,**H**,**J**,**L**,**N**,**P**) = 25 μm/×400.

Seven hours after inoculation with *C. perfringens* type A, the infected intestinal tissue was removed and using histological staining, we were able to visually detect pathological changes in the intestinal loop tissue. Macroscopically, the intestinal loop injected with *C. perfringens* type A was markedly distended, with thinning of the intestinal wall and a medium amount of gas in the intestinal lumen, which could be palpable and fluctuating in the intestinal tract. In addition, there was a small amount of reddish-brown viscous fluid in the intestine. Histologically, in the control group the intestinal villi were structurally intact (Figure 2A–D) and the intestinal tissue structure was clearly layered, with the mucosal layer, submucosal layer and muscular layer clearly visible (Figure 2I–L). In contrast, the loops of the *C. perfringens*-treated group (CP group) showed multiple apical epithelial cells of the villi were shed and necrotic (Figure 2E,F), with marked capillary dilation and haemorrhage, which are distinctive features of enteritis caused by *C. perfringens* type A infection (Figure 2G,H). In addition, we observed a proliferation of connective tissue in the submucosa (Figure 2M,N), with varying numbers of inflammatory cell infiltrates in between (Figure 2O,P). Collectively, these results demonstrate that the normal physiological structure of the intestine of deer is severely disrupted after inoculation with *C. perfringens* type A and that signs of enteritis, such as haemorrhage and inflammation, are evident.

### 3.2. C. perfringens Type A Destroys the Related Gene of Intestinal Barrier Immune System and Haematopoietic System

**Figure 3:**
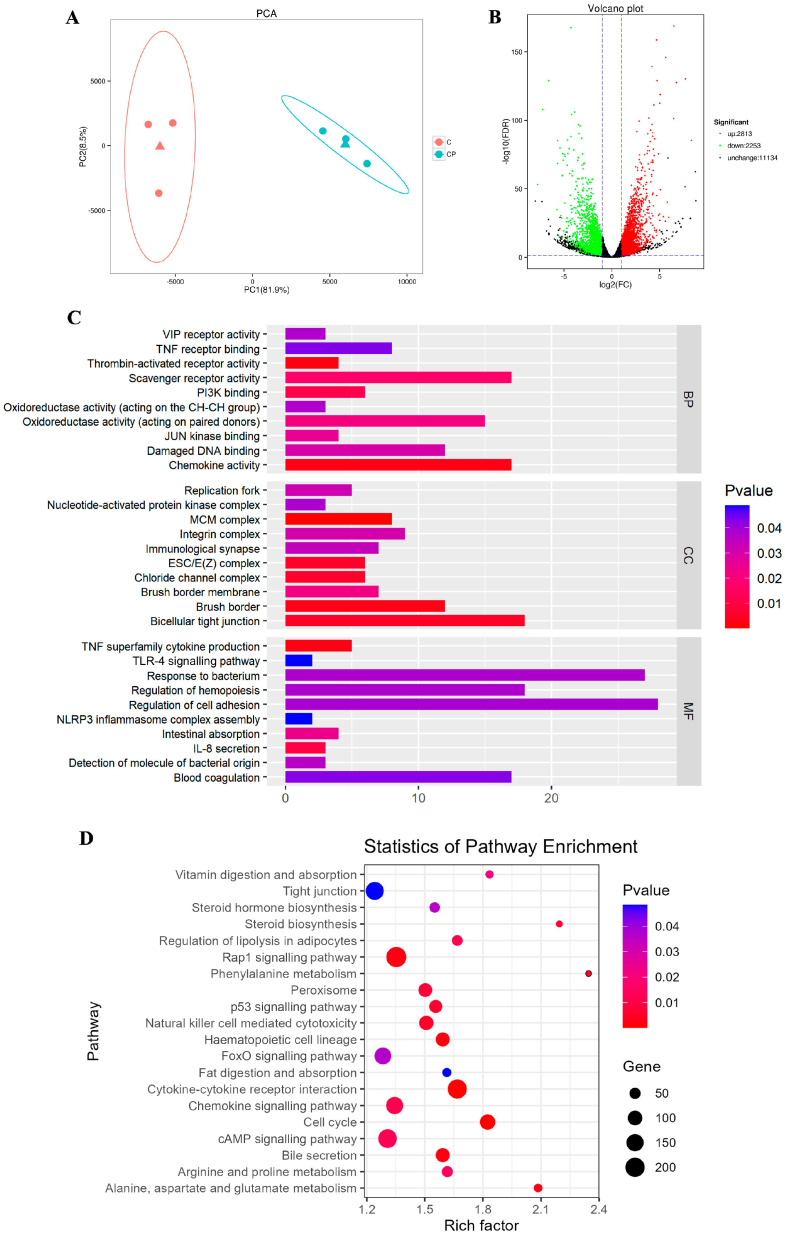
Transcriptional profile of the intestine after inoculation with *C. perfringens*. (**A**) Principal component analysis of the transcriptome (*n* = 3) of intestinal loops infected with *C. perfringens*. (**B**) Volcano plot shows gene expression analysis with upregulated genes in red and downregulated genes in green. (**C**) Bar graph shows GO pathway enrichment analysis of DEGs between intestines inoculated with or without *C. perfringens*. GO terms are classified into three categories: biological process, cellular component and molecular function. (**D**) The bubble map shows the pathway enrichment analysis by KEGG analysis of whether the intestine is inoculated with *C. perfringens* of DEGs. C = Control, CP = *C. perfringens*.

To investigate the molecular mechanisms of *C. perfringens* type A enteritis, we performed large-scale RNA sequencing of intestinal tissues from control and CP group. First of all, in the principal component analysis (PCA) scatter plot, we found that similar samples grouped, suggesting that the intra-group repeatability was relatively good, while there is a clear differentiation between the two groups (Figure 3A). By comparing the intestinal transcripts of the two groups, 5066 differentially expressed genes (DEGs) were identified, of which 2813 were upregulating DEGs and 2253 were downregulating DEGs (fold change > 2, q < 0.05) (Figure 3B).

To gain a comprehensive understanding of the effect of *C. perfringens* type A on intestinal genes, we performed gene ontology (GO) enrichment and Kyoto Encyclopedia of Genes and Genomes (KEGG) analysis of the DEGs (Figure 3C,D). Notably, the tight junction pathway was significantly enriched (*p* < 0.05) in the comparison of intestines inoculated with or without *C. perfringens* type A, including bicellular tight junctions and regulation of cell adhesion in the GO analysis and tight junctions in the KEGG analysis. Tight junctions regulate paracellular permeability and have a crucial role in preventing bacterial entry into the intestine by intracellular or paracellular means as well as promoting the composition of the intestinal epithelial barrier [20]. The significant enrichment of tight junctions reaffirmed that *C. perfringens* type A disrupts the fallow deer intestinal barrier and causes damage to the intestine of deer.

The results showed that these DEGs were also predominantly enriched in several biological processes and related pathways associated with immunity, oxidative stress and haematopoiesis, as revealed by the GO analysis. Among them, IL-8 secretion, immunological synaptic and chemokine activity are immune-related signalling pathways; oxidoreductase activity and scavenger receptor activity are signalling pathways associated with oxidative stress, regulation of haematopoiesis and blood coagulation; and thrombin-activated receptor activity is a signalling pathway associated with the haematopoietic system. In addition, GO analysis revealed that the pathways associated with *C. perfringens* type A infection such as response to bacterium, NLRP3 and TLR pathways were also found to be predicted to be activated (*p* < 0.05) (Figure 3C). Interestingly, the results of the KEGG analysis corresponded to the GO analysis, with DEGs enriched in the pathways of natural killer cell-mediated cytotoxicity, chemokine signalling pathways, peroxisome and haematopoietic cell lineages (Figure 3D).

The above results collectively suggest that enteritis induced by *C. perfringens* type A promotes intestinal barrier damage through multiple pathways, including suppression of the immune system, disruption of the haematopoietic system, promotion of intestinal oxidative stress and disruption of intestinal tight junctions.

### 3.3. C. perfringens Type A Affects the Intestinal Barrier Function by Reducing Intestinal Tight Junction Protein Expression

**Figure 4:**
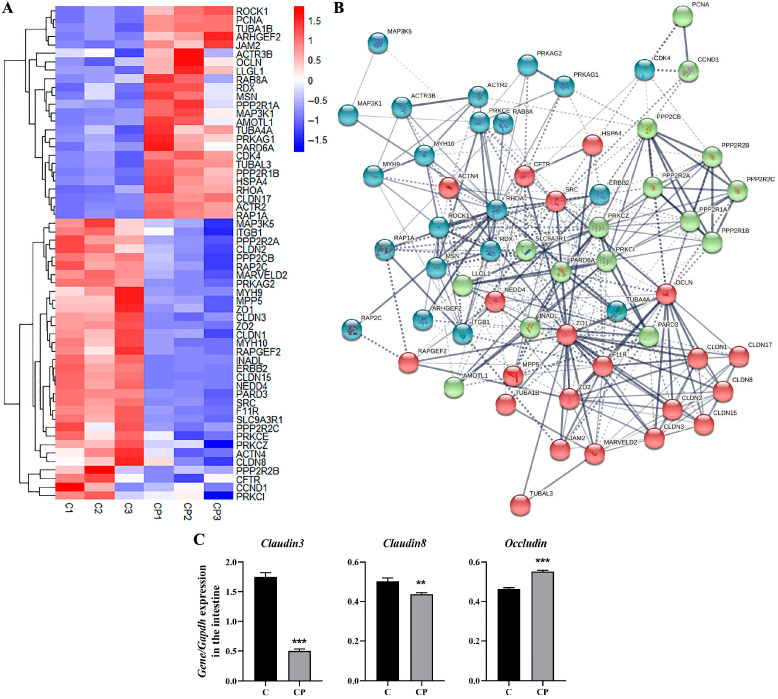
Genes significantly altered in the tight junction pathway in *C. perfringens*-infected intestines. (**A**) Heatmap showing tight junction genes with different expression. (**B**) Gene interaction network of genes that will vary in the tight junction pathway produced through the STRING website in the http://string-db.org. And, the genes were clustered, where the red circle proteins were mainly located in the intestine. (**C**) Changes in *Claudin3, Claudin8* and *Occludin* mRNA levels in the intestine of deer. All results are expressed as mean ± SEM. The level of statistical significance of all data was determined by independent samples *t*-test. * indicates significant differences compared to the control group, specifically ** *p* < 0.01 and *** *p* < 0.001.

To further investigate how *C. perfringens* type A disrupts the intestinal barrier of deer, we next performed further analysis of the transcriptome results. We found in the GO analysis that the PI3K pathway was enriched, whereas previous studies have shown that PI3K/AKT is further activated by tight junction proteins [21]. Furthermore, among the tight junction pathways activated by the GO analysis and KEGG analysis, the tight junction pathway was significantly enriched in both the GO and KEGG enrichment analysis. Therefore, we used a heatmap to further reveal possible pairs of DEGs in the tight junction pathway (Figure 4A). The results showed that the Claudin family showed a significant decrease in the CP group compared to the control group. Simultaneously, the genes obtained from differential gene heatmap analysis were subjected to protein interactions in STRING. And after hiding the disconnected nodes in the protein network, we found that all claudins, which are mainly distributed in the intestine, were altered and showed significant clustering (Figure 4B). This suggests that *C. perfringens* type A enters the intestine of deer via PI3K/AKT, disrupting the intestinal barrier by reducing the expression of tight junction proteins, which in turn promotes intestinal injury. To verify the correctness of the transcriptome results, we analyzed the mRNA levels of *Claudin3, Claudin8* and *Occludin* in the transcriptome and found that *Claudin3* and *Claudin8* expression both decreased, while *Occludin* increased (Figure 4C). These results were verified by quantitative real-time PCR (qPCR) and were consistent with the trends observed in the transcriptomic analysis.

### 3.4. C. perfringens Type A Can Be Recognized by TLR6 in the Intestinal Barrier of Deer

**Figure 5:**
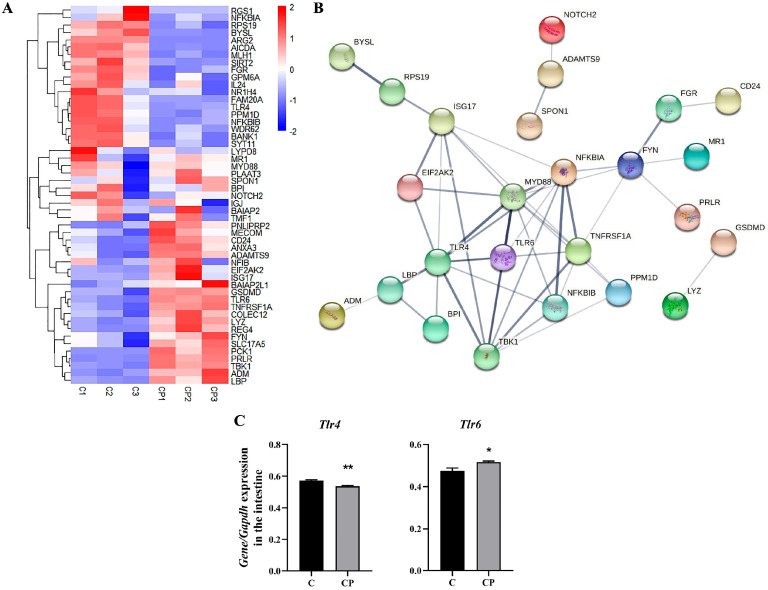
Genes altered in the pathway of response to bacterium in the *C. perfringens*-infected gut. (**A**) Heatmap showing differential expression of genes in response to bacterium. (**B**) A partial protein map of the gene interaction network of response to bacterium pathway generated through the STRING website, after removing proteins with lesser degrees of interaction, is available at http://string-db.org. (**C**) Changes in Tlr4 and Tlr6 mRNA levels in the intestine with *C. perfringens* infection. All results are expressed as mean ± SEM. The level of statistical significance of all data was determined by independent samples *t*-test. * indicates significant differences compared to the control group, specifically * *p* < 0.05 and ** *p* < 0.01.

To investigate how *C. perfringens* type A enters the intestine of deer, we analyzed the generated transcriptome data. We performed heatmap enrichment of genes related to the response to bacteria from the GO analysis to further reveal genes that may potentially be specifically responsive to *C. perfringens* type A (Figure 5A). Afterwards, the genes obtained from the differential gene heatmap analysis were subjected to protein interactions in STRING. And after removing the proteins with a lesser degree of interactions, the results reveal that *Tlr6* plays a key role in protein interactions in addition to being elevated in the heatmap (Figure 5B). Previous studies have shown that the ability of toll-like receptors (TLRs) to recognize bacterial cell wall components is thought to play an important role in bacterial–host interactions. And similarly, TLR6 is one of the TLRs capable of recognizing bacteria [22]. Therefore, we propose that *C. perfringens* type A is initially recognized by *Tlr6* upon intestinal entry. Conversely, we found that *Tlr4* showed a trend of decrease in the heatmap. In addition, the elevation of the TLR downstream gene Myd88, accompanied by downregulation of *Nfκbia* and *Nfκbib*, collectively demonstrate TLR6-mediated induction of inflammatory responses following pathogen recognition (Figure 5A). Analysis of the mRNA levels of related genes also showed a decrease in *Tlr4* and an increase in Tlr6 in the intestine in the CP group (Figure 5C).

### 3.5. Enteritis Caused by C. perfringens Type A Promotes the Expression of Immunoinflammation-Related Proteins

**Figure 6:**
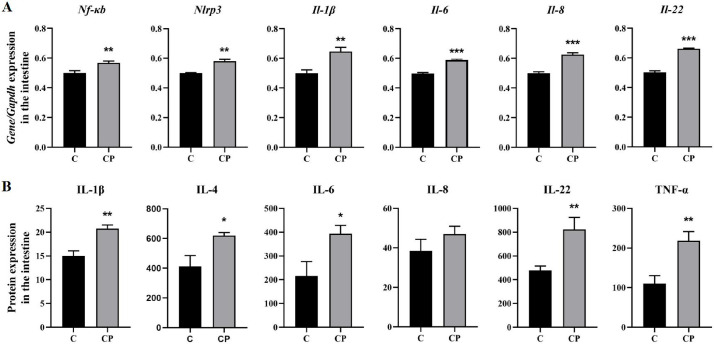
Changes in mRNA and protein of immune-related genes in the *C. perfringens*-infected intestine. (**A**) Changes in mRNA of Nf-κb, Nlrp3, Il-1β, Il-6, Il-8 and Il-22 in the *C. perfringens*-infected intestine. (**B**) Changes in protein of IL-1β, IL-4, IL-6, IL-8, IL-22 and TNF-α in the *C. perfringens*-infected intestine. All results are expressed as mean ± SEM. The level of statistical significance of all data was determined by independent samples *t*-test. * indicates significant differences compared to the control group, specifically * *p* < 0.05, ** *p* < 0.01 and *** *p* < 0.001.

Pathogens trigger an inflammatory process that damages the tight junctions, thereby disrupting the intestinal barrier [23]. Based on the results of the histological and transcriptomic analyses, it was known that *C. perfringens* type A inoculation triggered an inflammatory and immune response in the deer intestine after enteritis. To investigate, we performed mRNA and protein-level assays of immunoinflammation-related pathways by the Enzyme-linked Immunosorbent Assay (ELISA). The study showed that the TLR signalling pathway leads to activation of NF-κB, which in turn leads to upregulation of pro-inflammatory factors, such as IL-1β, 6 and TNF-α [24]. Therefore, we found that both genes and proteins of the interleukin family (IL-1β, IL-4, IL-6 and IL-22) were significantly upregulated in the CP group and that protein levels of IL-8 tended to increase in the CP group (Figure 6). In addition, the mRNA levels of both Nf-κb and Nlrp3 inflammasome were significantly elevated, demonstrating that *C. perfringens* type A triggers intestinal inflammation. The results suggest that *C. perfringens* type A damages the intestine and induces enteritis mainly by promoting immunoinflammation-related proteins.

### 3.6. Effect of C. perfringens Type A Infection on the Intestinal Antioxidant Capacity of Deer

**Figure 7:**
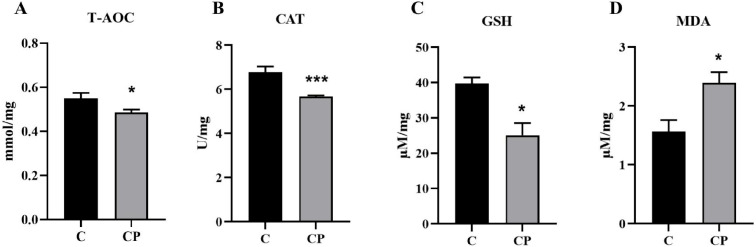
Effect of *C. perfringens* infection on antioxidant capacity in the intestine. Changes in intestinal antioxidant capacity following *C. perfringens* infection of the intestine were assessed by measuring T-AOC, CAT, GSH and MDA levels in the intestine using commercial kits. All results are expressed as mean ± SEM. The level of statistical significance of all data was determined by independent samples *t*-test. * indicates significant differences compared to the control group, specifically * *p* < 0.05 and *** *p* < 0.001.

Studies have shown that *C. perfringens* type A mediated an increase in reactive oxygen species (ROS) content and a decrease in cellular antioxidant capacity in intestinal epithelial cells [25]. This is supported by our transcriptome results for significant enrichment of oxidoreductase activity and scavenger receptor activity in the GO analysis and peroxisome pathway in the KEGG analysis. To assess the level of intestinal antioxidant capacity and to validate the results of transcriptome analysis, total antioxidant capacity (T-AOC), catalase (CAT), malondialdehyde (MDA) and glutathione (GSH) were measured in the intestine before and after *C. perfringens* type A infection using antioxidant kits. The T-AOC is widely used to assess the total antioxidant capacity of all antioxidants in a sample and reflects, to some extent, the total ability of the body to scavenge reactive oxygen species/nitric oxide synthase (ROS/NOS). Also, CAT and GSH both have antioxidant activity of substances, and conversely, MDA reflects the level of lipid oxidation. We found that T-AOC, CAT and GSH levels were significantly lower (Figure 7A–C) and MDA levels were significantly higher (Figure 7D) in the intestine inoculated with *C. perfringens* type A compared to the control intestine. The results indicated that the deer intestinal antioxidant enzyme activity was reduced and lipid oxidation was increased, and the total antioxidant capacity of the intestine was significantly decreased after inoculation with *C. perfringens*.

## 4. Discussion

*C. perfringens* type A is one of the main pathogens causing enteritis in various animals [26,27]. However, the network of genetic changes in the intestines of animals caused by *C. perfringens* type A is poorly understood due to the complexity of the intestinal transcriptome in pathological states [28]. This is a major obstacle to identifying the molecular mechanisms associated with *C. perfringens* type A-induced enteritis in deer. Here, we explored the structural and functional changes in the intestine caused by enteritis in deer intestinal infections of *C. perfringens* type A, by constructing an intestinal loop model. The results revealed that the normal structure of the deer intestine is strongly affected by *C. perfringens* type A infection, with significant downregulation of intestinal tight junction protein expression, contributing to inflammation and loss of intestinal epithelial barrier integrity. As shown in previous studies, TLR6 was activated in the intestinal tissues in response to bacteria in this experimental infection, promoting increased release of immune inflammatory markers [29]. In our study, the intestine of the deer also showed haemorrhage, consistent with enrichment of pathways associated with the haematopoietic system. Additionally, the upregulation of oxidative stress markers in the intestine and the downregulation of antioxidant capacity, along with decreased immune function and impaired barrier function, further confirm the negative effects of *C. perfringens* type A on intestinal health. By comparing *C. perfringens* type A-induced enteritis with the normal intestinal transcriptome, this experiment reveals how *C. perfringens* type A induces enteritis in deer through the immune system, the haematopoietic system, intestinal tight junction proteins and oxidative stress, providing a new target for the treatment of the disease.

Firstly, we replicated enteritis in deer by inoculating *C. perfringens* type A strains isolated from cases of deer enteritis into the intestinal loop. This model has been used extensively in the laboratory because of its ability to preserve the intact neural and vascular system of the intestinal environment [30,31,32]. Compared to the introduction of *C. perfringens* into the intestine by oral administration or enteral instillation [33], the enterocyclic model in ruminants can be studied in a single animal, thus reducing the number of experimental animals and facilitating experimenter handling. Nevertheless, all intestinal treatments were performed on the same deer, which limits the generalizability of the results and statistical inference. To address this limitation, our approach was guided by two key considerations: (1) given that deer are large animals, subject to stringent ethical constraints and resource acquisition challenges, this protocol specifically aimed to minimize animal use; and (2) the core objective was to directly compare the effects and potential interactions of different treatments on the intestines in real time within the same, strictly controlled physiological system. This design was essential to eliminate confounding effects arising from significant inter-individual variability.

Previous clinical trials have shown that *C*. *perfringens* type A is capable of causing intestinal disease in ruminants, rabbits and chicks. Infection of ruminants with *C*. *perfringens* type A may lead to diseases such as enteritis. The pathological changes in the intestinal tract are relatively complex, as follows: mild cicatricial inflammation in the small intestine, or haemorrhagic enteritis changes where the intestine is filled with fresh blood-like material, and in severe cases, lesions such as congestion and oedema in the large intestine may occur [1,34]. In addition, infection of ruminants with *C*. *perfringens* type A may cause acute or most acute cases with a short course and rapid death [5,35]. Compared to *C. perfringens* type A, the pathological signs of infection in the intestine with other types of *C. perfringens* can differ between different animals. When post-infected with *C. perfringens* type C, necrotic or necrohaemorrhagic lesions were observed in the small intestine and colon of horses [36], while in sheep and cattle, infection of sheep with *C perfringens* types B and C can lead to similar intestinal lesions, with enteritis manifesting as diffuse or multifocal haemorrhagic and necrotic enteritis, mainly in the ileum [37]. When piglets are infected with *C. perfringens* type C, pathological changes such as tissue necrosis and haemorrhage occur in the intestines, mainly in the jejunum, and in some cases in the ileum [38]. In the present study, deer intestines showed the hallmark pathological signs of enteritis, such as haemorrhage and necrosis (Figure 1). In addition, our results are consistent with previous reports that acute bacterial infections are primarily characterized by neutrophil infiltration [39]. In contrast, pathological analysis in this experiment revealed that *C. perfringens* type A infection of the intestine disrupts the original structure of the intestine, causing a large number of neutrophil aggregates and promoting an inflammatory response (Figure 1). It should be noted that the use of FTG medium as a control group rather than bacteria in this study may produce confounding effects.

As we all know, the tight junctions of the intestinal epithelial cells are an important part of the intestinal epithelial barrier, which limits paracellular permeability and is the front line of defence against bacterial invasion [40]. However, pathogenic microorganisms inhibit the expression of tight junction proteins such as claudins and occludins and disrupt the integrity of intestinal tight junctions to facilitate the entry of bacterial toxins into internal organs [41,42]. It is known that enterotoxins produced by *C. perfringens* type A act mainly through Claudin3 and Claudin4 due to the structural match of their binding domains [43]. Similarly, other tight junction proteins were altered directly or indirectly, affecting the normal state of the intestinal barrier [25,44]. In our study, Claudin3 and Claudin8 decreased and Occludin increased after *C. perfringens* treatment, suggesting that *C. perfringens*-induced enteritis can partially disrupt intestinal tight junction proteins and increase intestinal permeability (Figure 3A,C). Studies have shown that bacteria induce changes in tight junction proteins through PI3K/AKT, disrupting the integrity of the intestinal barrier and promoting further bacterial infection of the host, resulting in inflammatory responses [21]. In the same way, the significant enrichment of the PI3K binding pathway in the transcriptome results analysis in this study also indicated that *C. perfringens* disrupts tight junction proteins through the PI3K/AKT pathway and promotes intestinal damage (Figure 3B). Our data confirmed that deer intestinal infection with *C. perfringens* resulted in a significant reduction in intestinal tight junction proteins, leading to damage of the intestinal barrier, entry of bacterial toxins into the bloodstream and activation of the inflammatory response.

Research has shown that TLRs are critical for bacterial binding to intestinal epithelial cells and that *C. perfringens* toxins can amplify inflammatory responses and enhance virulence via TLR4 [45,46]. In addition, a study on the host immune response induced by *C. perfringens* infection in pig ileum indicated that, unlike the jejunum, *C. perfringens* was able to activate the TLR4/MyD88/NF-κB signalling pathway in the ileum, activating downstream immune-related cytokines [35,47]. However, our results revealed that TLR4 was significantly reduced in the CP group, while TLR6 was significantly increased (Figure 4A,C), possibly as a result of the specific differences between pigs and deer. Although the purpose and efficacy of TLR6 in the *C. perfringens*-infected jejunum remains to be determined, evidence suggests that TLR6 contributes to the maintenance of a balanced immune environment in intestinal diseases, including aiding the repair of damaged epithelial mucosa, managing intestinal inflammation and intestinal barrier integrity [48]. Dysregulation of TLR6 signalling has also been widely implicated in a range of diseases, such as ulcerative colitis and mild malaria [49]. It was shown that *C. perfringens* induces monocytes to produce TNF-α, which contributes to epithelial cell detachment [50,51]. Because of their pro-inflammatory and immunomodulatory functions, TNF-α and IL-8 play a key role in the pathogenesis of *C. perfringens*-induced enteritis [52,53]. Moreover, NLRP3 can also be upregulated by TLRs-induced NF-κB activation and leads to activation of the inflammatory response [54]. Our results demonstrated that *C. perfringens* can activate TLR6 to induce the synthesis of cytokines such as TNF-α, NLRP3 and interleukins through the MYD88/NF-κB pathway, promoting the development of intestinal inflammation and the activation of the immune system (Figure 4).

Oxidative stress is defined as the result of excessive production and accumulation of ROS in living organisms [55], which leads to structural cellular damage and results in irreparable oxidative damage and cell death [56]. Specifically, ROS targets the intestine and its cell membranes, causing lipid peroxidation, cell membrane disintegration and endothelial cell damage [57,58]. As mentioned earlier, the *C. perfringens* α-toxin is the most potent extracellular enzyme produced by *C. perfringens* and is capable of inducing ROS production and cytotoxicity [59]. In particular, it has been reported that after exposure to this toxin, a large amount of mitochondrial ROS was produced, leading to changes in the mitochondrial genome and function, further initiating immune patho-physiological mechanisms [60]. In experiments with LPS-induced intestinal epithelial cells, other studies have indicated that the NF-κB pathway is involved in promoting pro-inflammatory cytokine release and exacerbating oxidative damage [61]. For this reason, animals will scavenge the generated ROS to prevent oxidative stress through a variety of antioxidants, of which the intracellular CAT and GSH are key [62]. Similarly, we observed a decrease in T-AOC, CAT and GSH levels and an increase in MDA levels in the intestine infected with *C. perfringens*, which indicated that the intestinal antioxidant capacity was reduced (Figure 6).

Our GO and KEGG analysis showed significant activation of oxidative stress pathways, such as oxidoreductase activity and scavenger receptor activity and peroxisome (Figure 4C). Scavenger receptors are involved in the recognition and clearance of pathogens and serve multiple functions in lipid metabolism and oxidative stress, resisting elevated oxidative stress in cells due to lipid peroxidation [63]. This validates the antioxidant assay results and reveals oxidative stress in the intestine due to the damaged defence mechanisms of the antioxidant system. Thus, it is shown that there is a combination of multiple factors that directly or indirectly accelerate the disruption of intestinal tight junctions and *C. perfringens* type A-induced enteritis in the present study. However, the effects of different *C. perfringens* toxins on deer are not addressed in the study, which may affect the generalizability of this work. Future work should focus on the effect of different *C. perfringens* typing on deer intestine and further verify their molecular mechanisms.

## 5. Conclusions

The present study provides a basis for elucidating a series of responses in the deer intestine following *C. perfringens* type A inoculation. Here, TLR6 was activated in intestinal tissues in response to bacteria, promoting the increased release of immune markers. At the same time, upregulation of oxidative stress markers and downregulation of antioxidant capacity in the intestine led to decreased immune function and impaired barrier function. Infection also leads to downregulate tight junction proteins via the PI3K/AKT pathway, contributing to inflammation and loss of intestinal epithelial barrier integrity and intestinal haemorrhaging. Our study provides mechanistic insights for the molecular mechanisms of *C. perfringens* type A-induced enteritis in deer at the transcriptome level and provides a broader guide for exploring *C. perfringens* type A-induced enteritis and other intestinal diseases.

## Figures and Tables

**Table 1:** Primers used in this study.

Primer	Direction	Sequences (5′ to 3′)
*Claudin3*	Forward	GAGGGCCTGTGGATGAACTG
*Claudin3*	Reverse	GAAGACGGCCAGTAGGATGG
*Claudin8*	Forward	AAAACTGCTCTCCCTCGGTG
*Claudin8*	Reverse	GGCGTAGGTAGCCATTCTCC
*Occludin*	Forward	AATAGTGAACGCCGTCCTGG
*Occludin*	Reverse	GGTCTGGGCAGTTGGATTGA
*Tlr4*	Forward	CTCCTGCCTGAGATCCGAGA
*Tlr4*	Reverse	AGGTCCAGCATCTTGGTTGTT
*Tlr6*	Forward	TGCTGATTACAGTGGATGTTGTG
*Tlr6*	Reverse	AACTGACCCCAAGGCTGATG
*Nf-κb*	Forward	TTGGCAACAACACTGACCCT
*Nf-κb*	Reverse	CCATGGGTACACCCTGGTTC
*Nlrp3*	Forward	TTCCCATCAGTGCTGCTTCA
*Nlrp3*	Reverse	GGCCAGAATTCACCAACCAG
*Il-1β*	Forward	CTGTGGCCTTGGGTATCAGG
*Il-1β*	Reverse	GCCACCTCTAAAACGTCCCA
*Il-6*	Forward	ACGAGTGGGTAAAGAACGCA
*Il-6*	Reverse	GGAATGCCCAGGAACTACCA
*Il-8*	Forward	GACCCCAAGGAAAAGTGGGT
*Il-8*	Reverse	CCACACAGTACTCAAGGCACT
*Il-22*	Forward	ACCCTGAAACGTGAATGTGC
*Il-22*	Reverse	AGGACTGTGGAGTTTGGCTT
*Gapdh*	Forward	GAGCACGAGAGGAAGAGAGTT
*Gapdh*	Reverse	TTGGGGATGGAAACTGTGGA

Among these, *Gapdh* was used as the housekeeping gene.

## Data Availability

The data presented in this manuscript and its Supplementary Materials are available from the corresponding author upon request.

## Supplementary Materials

The following supporting information can be downloaded at: https://www.mdpi.com/article/10.3390/genes16080949/s1.

## Abbreviations

The following abbreviations are used in this manuscript:

*C. perfringens*	*Clostridium perfringens*
CAT	Catalase
CPA	*C. perfringens* α-toxin
DEG(s)	Differentially expressed gene(s)
ELISA	Enzyme-linked immunosorbent assay
FTG	Fluid thioglycollate
GO	Gene ontology
GSH	Glutathione
KEGG	Kyoto Encyclopedia of Genes and Genomes
MDA	Malondialdehyde
PC	Phosphatidylcholine
PPI	Protein–protein interaction
qPCR	Quantitative real-time PCR
ROS/NOS	Reactive oxygen species/nitric oxide synthase
SM	Sphingomyelin
T-AOC	Total antioxidant capacity
TLRs	Toll-like receptors
